# Hydrothermal carbonisation of peat-based spent sorbents loaded with metal(loid)s

**DOI:** 10.1007/s11356-019-05653-6

**Published:** 2019-06-16

**Authors:** Alfreda Kasiuliene, Ivan Carabante, Prosun Bhattacharya, Jurate Kumpiene

**Affiliations:** 1 Department of Civil, Environmental and Natural Resources Engineering, Lulea University of Technology, SE-97187 Lulea, Sweden; 2 Department of Sustainable Development, Environmental Science and Engineering, Royal Institute of Technology, Teknikringen 76, SE-100 44 Stockholm, Sweden

**Keywords:** Metals, Adsorption, Thermal treatment, Iron-peat, Post-sorption management, HTC

## Abstract

Hydrothermal carbonisation (HTC) is a wet and relatively low-temperature process where, under autogenous pressures, biomass undergoes a chain of reactions leading to the defragmentation of organic matter. As well as its other uses (e.g. for producing low-cost carbon-based nano-compounds), HTC is utilised for the treatment of wet wastes, such as manure and biosludge. This study aimed to determine if hydrothermal carbonisation is a feasible treatment method for spent sorbents that are highly enriched with arsenic, chromium, copper, and zinc. The chemical properties of hydrochar and process liquid were evaluated after HTC treatment, where peat-based spent sorbents were carbonised at 230 °C for 3 h. Analysis of Fourier transform-infrared spectra revealed that during HTC, the oxygenated bonds of ethers, esters, and carboxylic groups were cleaved, and low-molecular-weight organic fragments were dissolved in the process liquid. A large fraction of arsenic (up to 62%), copper (up to 25%), and zinc (up to 36%) were transferred from the solids into the process water. Leaching of these elements from the hydrochars increased significantly in comparison with the spent sorbents.

## Introduction

Contamination of groundwater and surface waterbodies due to natural and anthropogenic activities has been reported worldwide. By their nature, contaminants may be organic or inorganic. Inorganic contaminants include metals, such as chromium (Cr), copper (Cu), and zinc (Zn), and metalloids, such as arsenic (As). These metal(loid)s are commonly associated with pollution and toxicity problems (Lafa et al. 2015; Küpper and Andresen 2016). A common method to remove metal(loid)s from contaminated water is to adsorb them onto a reactive media and then separate them from the solution. This process can effectively be applied under a wide range of pH conditions (Mohan and Pittman 2007; Lim et al. 2013). The sorbents used can be of mineral or biological origin including zeolites, industrial by-products, polymeric materials, and biomass including agricultural, forestry, and fishery wastes (Kurniawan et al. 2005). Although continuous improvements are being made to achieve higher removal efficiencies, little attention has been paid to the management of spent sorbents loaded with adsorbed metal(loid)s. If sorbents are used to adsorb oxyanion-forming elements, such as As, regeneration techniques are limited and/or expensive (Verbinnen et al. 2015) and the recovery of As is not preferred due to a limited market. Thus, the predominant management choice for such spent sorbents is landfilling. Anaerobic conditions and a reducing environment, as is common in landfills, can increase the mobility of redox-sensitive elements, like As, thus creating leachate treatment problems (Corvin et al. 1999). To overcome this issue, treatments for spent sorbents are required either as an alternative to landfilling or to reduce metal(loid) leaching when waste is placed in landfill.

Thermal treatment plays a key role in modern waste management systems (Lombardi et al. 2015). Combustion, gasification, and pyrolysis of combustible wastes are the most common processes available for thermochemical conversion. As an alternative to these processes, hydrothermal carbonisation (HTC) opens up the possibility to treat wet waste streams, without energy-intensive drying before or during the treatment. Hydrothermal carbonisation is a wet and relatively low-temperature (180–350 °C) process where, under autogenous pressures, biomass undergoes a chain of reactions including hydrolysis, dehydration, decarboxylation, condensation, polymerisation, and aromatisation (Wikberg et al. 2015). This technology firstly was developed to convert biomass into a more coal-like material. Recently, HTC has received attention as a method for producing low-cost carbon-based nano-compounds used for soil fertilisation, amelioration, and carbon sequestration (Libra et al. 2011). Wet manure and biosludge are common waste streams treated by HTC. The method offers disinfection, odour improvement, and toxicity reduction of many organic contaminants present in manure and biosludge (Heilmann et al. 2014). Recent studies from Lucian and Fiori (2017) and Volpe et al. (2018) showed that HTC of biomass waste, such as olive oil mill waste, followed by hydrochar pelletisation could represent an economic and sustainable approach for the production of solid-densified bio-fuels.

The composition of the raw material intended for HTC has a decisive influence on the final product. The application of HTC for complex waste streams, such as sorbents loaded with metal(loid)s, is underexplored. Some studies have reported the concentrations of metal(loid)s in biomass and associated hydrochars (Reza et al. 2013) or have at least included some discussion of their occurrence (Libra et al. 2011; Reza et al. 2014). However, biomass usually contains low levels of metal(loid)s, such as mercury (Hg), lead (Pb), As, Cr, Cu, Zn, or selenium (Se), and, as such, does not necessarily pose environmental risk (Reza et al. 2013). In contrast to clean biomass, spent sorbents contain significantly higher concentrations of contaminants. The behaviour of metal(loid)s under HTC conditions is not fully understood yet and, consequently, further utilisation of resulting hydrochars as soil additives is restricted. Yoshida and Antal (2009) showed that during flash carbonisation, inorganic contaminants with high boiling points (Pb, cobalt (Co), nickel (Ni), Cu, and Zn) remain in the hydrochar, whereas those with low boiling points (As, Hg, Cd, and selenium (Se)) are prone to elution. In addition, during HTC, the degradation of organic matter and the redistribution of organic groups can lead to the transfer of inorganic components, including contaminants, from solids into the process water (Krylova and Zaitchenko 2018). Therefore, it is reasonable to expect that HTC can be used to remove hazardous metal(loid)s from spent sorbents so that hydrochars can be used as soil ameliorants without the risk of secondary pollution.

The aim of the study was to determine the feasibility of HTC as a treatment method for peat-based spent sorbents (loaded with As, Cr, Cu, and Zn) to obtain a reusable hydrochar fraction. For this, the chemical properties of the hydrochar and the process liquid were evaluated after HTC treatment.

## Materials and methods

### Obtaining spent sorbent

The following two peat-based sorbents were used in this study: (1) heat-treated peat and (2) heat-treated peat coated with ferric ferrous hydrosol. Details of the sorbent preparation method and its efficiency can be found in Kasiuliene et al. (2019). The sorbents were mixed with metal(loid) solution at a liquid to solid (L/S) ratio of 4 and were left to dry at room temperature. The solution contained 1 g L^−1^ of As and 4 g L^−1^ of Cr, Cu, and Zn. This was prepared by dissolving analytical grade chemicals, namely NaH_2_AsO_4_ (Honeywell Riedel-de Haën AG, 99%), K_2_Cr_2_O_7_ (VWR International, 99.9%), CuCl_2_ · 2H_2_O (Merck, 99%), and ZnCl_2_ (Merck, 98%), in ultra-pure water. Total metal(loid) concentrations in the spent sorbents were determined using inductively coupled plasma optical emission spectrometry (ICP-OES) (Optima 8300, Perkin-Elmer) after wet digestion with *aqua regia* in a microwave oven (CEM Mars 5) at 190 °C. Prior to ICP-OES analysis, subsamples were filtered through 0.45-μm cellulose filters. Detection limits were as follows: 0.002 mg L^−1^ for As, 0.001 mg L^−1^ for Cr and Cu, 0.014 mg L^−1^ for Fe, and 0.002 mg L^−1^ for Zn. Hereafter, the spent peat sorbent is referred to as ‘peat’, while the spent iron-peat sorbent is referred to as ‘iron-peat’.

### Hydrothermal carbonisation

Triplicates of peat and iron-peat were placed into Teflon vessels and mixed with ultra-pure water at L/S = 5. Sealed vessels were heated to 230 °C at a rate of ~ 1.75 °C min^−1^ using a CEM Mars 5 microwave oven. The target temperature was maintained for 3 h. After cooling down, vessels were dismantled and the contents were filtered through 10-μm pure cellulose filters. The solids remaining on the filter were rinsed with ultra-pure water at L/S = 5. Hereafter, the solids obtained from the peat and iron-peat are referred to as ‘P-hydrochar’ and ‘IP-hydrochar’, respectively, and the process liquid—including the main filtrate and the rinsing water—is referred to as ‘P-liquid’ and ‘IP-liquid’, respectively.

### Characterisation of the process liquid

The electrical conductivity (EC) (CDM 10, Radiometer Copenhagen), pH (pH 340, WTW), and redox potential (Eh) (CDM 10, Radiometer Copenhagen) of the process liquid were measured after being separated from the hydrochars. Density was measured as the mass to volume ratio. Subsamples were analysed with ICP-OES for metal(loid)s. A total organic carbon analyser (TOC-L series, Shimadzu) was used to determine the dissolved carbon (DC) content of the process liquid. The detection limits for dissolved organic carbon (DOC) and for dissolved inorganic carbon (DIC) were 4 μg L^−1^.

### Characterisation of the spent sorbents and hydrochars

The air-dried spent sorbents and hydrochars were analysed for total solids (TS) and ash content following standard procedures (ISO 11465:1993). Their calorific values were determined using a combustion calorimeter (IKA C 200). Ultimate analysis for carbon (C), hydrogen (H), nitrogen (N), sulphur (S), and oxygen (O) content was carried out using a CHNS-O elemental analyser (Eurovector EA3000).

Batch leaching tests (L/S = 10) were carried out following standard procedures (EN 12457/2 2002). Briefly, triplicates of each sample were leached with ultra-pure water for 24 h using an end-over-end rotator, which were then filtered through 0.45-μm nitrocellulose filters and immediately analysed for EC, pH, and Eh. Metal(loid) content was determined with ICP-OES.

A three-step sequential extraction protocol (EUR 19775 EN) proposed by the EC Standards, Measurements and Testing Programme was applied to the spent sorbents and hydrochars. The protocol is described in detail by Sutherland (2010). Briefly, the exchangeable fraction was obtained after extraction for 16 h with 0.11 M acetic acid (Merck, 100%) solution; the reducible fraction was obtained after extraction for 16 h with 0.1 M hydroxylammonium chloride (Merck, 99%) solution at pH 2.0; the oxidisible fraction was obtained after a 3-h step-wise extraction with hydrogen peroxide (Merck, 35%) in a heated water bath at 85 °C, followed by a 1-h extraction with 1.0 M ammonium acetate (VWR International, 98.6%) solution at pH 2. The sequential extraction was finished by extracting the residual fraction with *aqua regia* at 190 °C for 10 min. The extractions were performed in triplicate and the extracts were filtered through 0.45-μm nitrocellulose filters, acidified (except the residual fraction), and analysed with ICP-OES.

### Identification of organic groups

Fourier transform-infrared (FT-IR) spectroscopy data on the finely powdered spent sorbents, their respective hydrochars, and the process liquids were collected using a Frontier FT-IR spectrometer (Perkin-Elmer). The FT-IR spectrometer was equipped with a DTGS (deuterated triglycine sulphate) detector and the samples were probed using a single reflection UATR diamond/Zn Se. The spectra were obtained from 50 co-added scans with a resolution of 4 cm^−1^ and a zero-filling factor of 2.

### Statistical analysis

The data were processed using an analysis of variance (ANOVA) test using Minitab 18 software. A post hoc two-sample *t* test (*p* > 0.05) was applied to differentiate between sample means.

## Results and discussion

### The main characteristics of HTC products

Initially, 8.0 ± 0.0 g TS of peat and iron-peat were used for carbonisation. After the experiment, 4.9 ± 0.2 g and 5.7 ± 0.1 g of P- and IP-hydrochars were recovered, respectively. Thus, 36% and 25% of solids from peat and iron-peat were transferred to the process liquid and gas phase.

The main properties of P- and IP-liquids are given in Table 1. Both process liquids had a high content of DOC. This was an expected outcome because due to increasing temperature as well as pressure, biomass is more accessible to water, which leads to the degradation of the physical biomass structure (Allen et al. 2001). Furthermore, a slightly acidic pH, as determined in the process liquids, was a likely result of low-molecular-weight organic acids (e.g. acetic and lactic acids) that are usually produced during biomass hydrolysis (Berge et al. 2011). A high content of DOC led to low Eh values and density values below unity, while EC values were up to eight times higher in comparison with the EC of drinking water. In general, the more water that is provided for the HTC process, the greater the C loss per mass unit of raw material. This not only lowers the yield of hydrochar but also results in a concentrated process liquid that requires further treatment (Libra et al. 2011). Bearing in mind that peat and iron-peat were loaded with As, Cr, Cu, and Zn, elevated concentrations of these elements were expected in the process liquid. To avoid large volumes of the (possibly contaminated) process liquid being produced, the L/S ratio was kept low (L/S = 5) to ensure the prerequisite of dry solids being submerged in water. Theoretically, the amount of water that is necessary to ensure carbonisation reactions is very small and some authors even propose that the water produced during deoxygenation reactions with organic matter can be sufficient for hydrolysis (Landais et al. 1994). If spent sorbents are to be hydrothermally carbonised directly after adsorption (with a moisture content up to 70%), the addition of water, and thus the production of process liquid products, could be minimised.

**Table 1:** Properties of the process liquids (average ± standard deviation, *n* = 3)

Analytes	P-liquid	IP-liquid
pH	5.9 ± 0.3	5.7 ± 0.1
EC, mS cm^−1^	9.8 ± 1.2	5.5 ± 0.5
Eh, mV	25.8 ± 8.1	14.6 ± 10.6
Density, g L^−1^	956 ± 14	952 ± 10
DC, mg L^−1^	7046 ± 228	7561 ± 397
DOC, mg L^−1^	7038 ± 223	7558 ± 396
IC, mg L^−1^	4.6 ± 1.3	3.0 ± 1.3

Table 2 shows the contents of total solids and ash, the results of the ultimate analysis, and the calorific values of the spent sorbents and their respective hydrochars. Solid yield (mass ratio of hydrochar to raw material on a dry weight basis) from peat was 64% and the yield from iron-peat was 75%. Both of these values were within the range of yields reported in other studies (Funke and Ziegler 2010; Libra et al. 2011; Reza et al. 2013; Krylova and Zaitchenko 2018); yields were in the range of 39 to 90%. Recent study from Lucian et al. (2018) suggests that formation of solids due to back-polymerisation of the organic molecules from the process water can further increase the hydrochar yield at higher temperatures of around 260–280 °C. However, varying experimental conditions (i.e. temperature, L/S ratio, reaction time, raw materials, their particle sizes, and pH of the media) do not allow direct comparison. Many researchers use the term ‘reaction severity’ which refers to higher temperatures and/or longer reaction times. With increasing reaction severity, hydrochar is produced with a high C content but at low yield. First reactions involved in HTC are observed already at 100 °C, but for substantial hydrolysis, a temperature of about 180 °C is necessary. Even higher temperatures (about 220 °C) are required to hydrolyse cellulose (Funke and Ziegler 2010). Therefore, slightly higher than the latter temperature was chosen for the HTC experiment in this study (230 °C) to ensure a complete hydrolysis of the main peat constituents. Typical published reaction times vary between 1 and 72 h. However, the effects of the reaction time for HTC are largely unknown. A reaction time of 3 h in this study was chosen bearing in mind practical aspects in case the experiment would be scaled-up where long reaction time would mean additional costs. Shorter reaction time and/or lower temperature could imply incomplete carbonisation.

**Table 2:** Properties of the spent sorbents and hydrochars (average ± standard deviation, *n* = 3)

Analytes	Peat	P-hydrochar	Iron-peat	IP-hydrochar
Total solids, %	89.2 ± 0.1	96.3 ± 0.6	93.4 ± 0.1	98.4 ± 3.6
Ash content, %	9.9 ± 0.3	13.7 ± 4.4	15.4 ± 0.2	18.2 ± 0.1
Calorific value, MJ kg^−1^	19.7 ± 1.2	29.5 ± 2.7	18.8 ± 0.8	28.9 ± 8.7
C, %	41.9 ± 0.7	60.5 ± 0.1	42.6 ± 0.5	55.2 ± 0.3
H, %	6.0 ± 0.1	5.6 ± 0.1	5.6 ± 0.1	5.3 ± 0.2
N, %	2.6 ± 0.1	3.1 ± 0.1	2.6 ± 0.1	2.8 ± 0.1
O, %	37.7 ± 0.8	22.6 ± 0.5	37.0 ± 0.1	24.5 ± 0.4
S, %	< 1	< 1	< 1	< 1

After the HTC treatment, the total solids and ash content increased as well as the calorific value. The calorific value of P-hydrochar was 33% higher than peat and that of IP-hydrochar was 35% higher than iron-peat. The hydrochars had significantly lower O/C and H/C ratios compared with the spent sorbents. The O/C ratio dropped from 0.86–0.89 to 0.37–0.44, while H/C dropped from 0.13–0.14 to 0.09. The contents of S and N were little affected by carbonisation.

Gases also evolve during HTC, and yet gas measurements are seldom reported. It is often restated that (i) carbon dioxide is the main gas evolving during HTC followed by minor fractions of carbon monoxide and methane and (ii) gaseous phases increase with increasing reaction temperature. Depending on the experimental setup, gas formation can scatter between 3 and 20% of the total carbonisation products (Funke and Ziegler 2010; Reza et al. 2013; Krylova and Zaitchenko 2018).

### FT-IR spectra

**Fig. 1:**
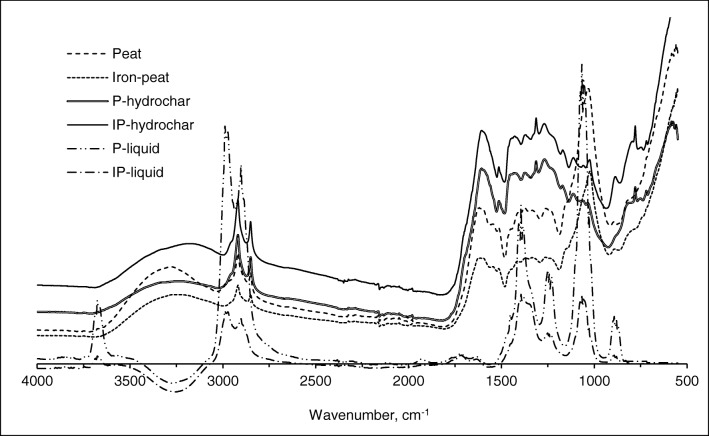
The FT-IR spectra on spent sorbents and their respective hydrochars and process liquids

Figure 1 shows the FT-IR spectra of the studied spent sorbents, hydrochars, and process liquids. Spectra of the spent sorbents and their respective hydrochars were similar and exhibited variable relative intensities typical of humic-like materials (Senesi and Steelink 1989). A wide band between 3600 and 3200 cm^−1^ was ascribed to the stretching of O–H bonds and the two distinct absorption bands between 3000 and 2800 cm^−1^ were attributed to the asymmetric stretching of the aliphatic C–H bonds of alkenes (3050 cm^−1^) and alkanes (2950 cm^−1^), respectively. The absorption band at 1600 cm^−1^ was assigned to C=O stretching of carbonyl functional groups. The absorption bands between 1600 and 1450 cm^−1^ stemmed either from C=C vibrations in aromatics or from C=O vibrations in carboxylic (COO–) groups. The absorption bands at approximately 1300 cm^−1^ were assigned to C–N vibrations of amines and amides, and to nitro compounds (NO_2_). The absorption band observed at 1040–1070 cm^−1^ was assigned to C–O stretching, which is representative of ether, ester, and carboxylic functional groups.

Compared with the spent sorbents, the hydrochar samples exhibited higher relative intensities of absorption bands assigned to O–H, C–H bonds, C=O, C=C, and C–N bonds (4000–1100 cm^−1^). In contrast, C–O bands assigned to ethers, esters, and carboxylic groups (1070–1040 cm^−1^) showed higher absorbance in spectra from the spent sorbents prior to HTC. According to Funke and Ziegler (2010), during HTC, hydrolytic reactions break ester and ether bonds. In addition, carboxylic groups can be rapidly eliminated above 150 °C, yielding carbon dioxide and carbon monoxide. This is in line with the spectra of the process liquid samples. A distinctive band at 3650 cm^−1^ was assigned to O–H stretching bonds in monomeric alcohols and carboxylic acids. Two typical bands between 3000 and 2800 cm^−1^ were associated with aliphatic C–H groups and indicated the presence of low-molecular-weight degradation fragments in the process water. A band at approximately 1400 cm^−1^ was associated with C=O stretching of esters, while two sharp bands at 1250 cm^−1^ and 1050 cm^−1^ resulted from C–O stretching of ethers and carboxylic groups. In general, the results obtained from the FT-IR spectra support those published elsewhere (e.g. Funke and Ziegler 2010) showing that the cleavage of easily oxygenated groups (i.e. ester, ether, and carboxylic groups) results in the presence of low-molecular-weight compounds (i.e. monomeric alcohols and carboxylic acids) in the process water.

### Behaviour of metal(loid)s

Data for metal(loid) behaviours with respect to the evolvement of gases during HTC are scarce. In this study, it was assumed that the maximum HTC temperature (230 °C) was too low to initiate volatilisation of As, Cr, Cu, and Zn. Therefore, all metal(loid) content should have been distributed between the hydrochars and process liquids.

**Fig. 2:**
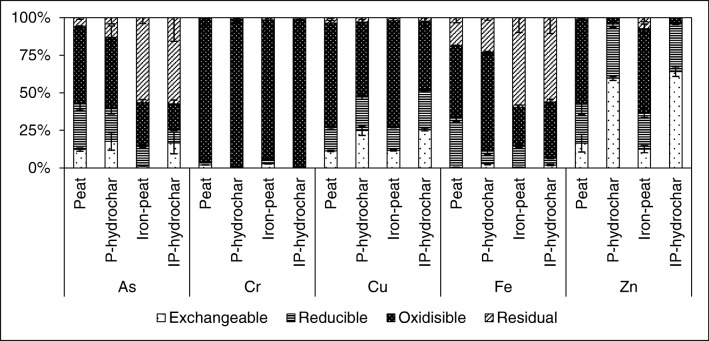
Metal(loid) fractionation in the spent sorbents and hydrochars. Error bars represent the standard deviation of the mean (*n* = 3)

Table 3 shows the metal(loid) concentrations in the leachates after the standardised batch leaching test along with the spent sorbents and hydrochars; the total metal(loid) concentrations and the amounts that were leached out (%) are shown as well. The average pH, EC, and Eh values in the leachates are presented in Table 4. The metal(loid) fractionation in the spent sorbents and hydrochars is shown in Fig. 2.

**Table 3:** Total metal(loid) concentrations (mg kg^−1^) in the spent sorbents and hydrochars, and concentrations (mg kg^−1^) in the leachates (average ± standard deviation, *n* = 3), and percentage of the total concentrations

Analytes	Peat	%	P-hydrochar*	%	Iron-peat	%	IP-hydrochar*	%
Total As Leachate As	411 ± 52 29.4 ± 0.5	7.1	288 ± 145 13.1 ± 1.3	4.6	993 ± 50 0.53 ± 0.16	0.1	700 ± 31 41.5 ± 3.1	5.9
Total Cr Leachate Cr	3673 ± 215 8.9 ± 0.1	0.2	3324 ± 183 0.01 ± 0.00	0.01	3821 ± 114 11.6 ± 0.2	0.3	3299 ± 112 0.01 ± 0.00	0.1
Total Cu Leachate Cu	3697 ± 76 27.9 ± 0.6	0.8	3227 ± 72 111 ± 6	3.5	3795 ± 58 39.9 ± 0.5	1.1	3348 ± 135 135 ± 3	4.0
Total Fe Leachate Fe	20,133 ± 1593 3.4 ± 0.2	0.02	14,965 ± 957 0.80 ± 0.01	0.01	63,295 ± 2659 38.5 ± 0.3	0.1	57,104 ± 6453 0.80 ± 0.01	0.01
Total Zn Leachate Zn	3728 ± 92 6.9 ± 0.4	0.2	3493 ± 127 222 ± 6	6.4	2823 ± 69 32.2 ± 1.7	1.1	2802 ± 98 272 ± 1	9.7

*Sum of concentrations from different fractions

**Table 4:** pH, EC, and Eh in the leachates after the standardised batch leaching test (average ± standard deviation, *n* = 3)

Analytes	Peat	P-hydrochar	Iron-peat	IP-hydrochar
pH	4.7 ± 0.1	5.6 ± 0.2	5.3 ± 0.1	5.6 ± 0.1
EC, μS cm^−1^	170 ± 11	810 ± 300	171 ± 32	690 ± 120
Redox, mV	250 ± 21	185 ± 2	280 ± 1	191 ± 1

#### Arsenic

The concentration of As in iron-peat was 2.4 times higher than in peat. We have previously reported that the adsorption of As increases by several times when peat is coated with Fe hydroxides (Kasiuliene et al. 2018, 2019). The improved efficiency of the iron-peat sorbent is attributed to a greater Fe content and a larger specific surface area. In this study, despite the high loads, only 0.1% of the adsorbed As leached from the iron-peat. In contrast, about 7% of the As leached from the peat, which was above the limit (25 mg kg^−1^) for waste being accepted at landfills for hazardous waste. Peat had a larger fraction of exchangeable As (12%) and a smaller fraction of residual As (6.2%) in comparison with iron-peat, where only 1.2% of all of the As was exchangeable and more than 55% was in the residual fraction (Fig. 2). When HTC was used as a treatment method for the spent sorbents, a large fraction of As was transferred to the process liquid; approximately 62% of the As was found in the P-liquid and approximately 35% was found in the IP-liquid. The leaching of As from the P-hydrochar was lower in comparison with the leaching from peat, 4.6% versus 7.1% respectively. However, the exchangeable As fraction in the P-hydrochar was actually higher than in the peat. Leaching from the IP-hydrochar was more than 100 times higher than from iron-peat, which coincided with a significant gain in the exchangeable fraction.

The interactions between As, Fe, and organic matter are complex and the effect of increasing temperature and pressure during HTC might have induced several reactions, which were possibly responsible for the increased leaching of As. Firstly, the oxidisible fraction of Fe in both hydrochars increased, whereas the reducible fraction decreased (Fig. 2). Such changes could be an indication that after HTC, Fe became strongly bound to organic matter, and the consequent competition for Fe sorption sites resulted in increased leaching of As (Cornell and Schwertmann 2004). The existence of stable Fe-organic matter complexes that are non-reactive towards As was also confirmed by Sundman (2014) and Sundman et al. (2015). Secondly, As is a redox-sensitive element. The redox potential determined in the process liquids after HTC was about 15–26 mV (Table 1), while Eh in the spent sorbent leachates after standardised batch leaching (Table 4) was 250–280 mV. During HTC, the concentration of dissolved O_2_ was almost zero due to increasing reaction severity, so it is possible that As(V) could have been reduced to As(III) and released into the process liquid. This could explain why the reducible As fraction became smaller in the hydrochars. McNeill et al. (2002) also reported speciation changes from As(V) to As(III) as an adverse consequence of water boiling.

#### Chromium

The total content of Cr was similar in both spent sorbents (Table 3). The limit for Cr content for the acceptance of wastes at hazardous landfills is 10 mg Cr kg^−1^. Leaching of Cr from peat was below this limit, while that of iron-peat was slightly above the limit. After HTC, > 99% of Cr remained in the hydrochars. The sequential extraction revealed that due to HTC, exchangeable and reducible fractions of Cr disappeared and all Cr in hydrochars was bound to organic matter (Fig. 2). A likely reason for this is that under reducing conditions, chromate Cr(VI) was reduced to Cr(III) and, under favourable pH conditions of 5.0–5.5 (Table 4), this resulted in the effective adsorption onto organic matter (Balan et al. 2009). Due to the HTC treatment, the leaching of Cr from both hydrochars was minimal.

#### Copper and zinc

Both spent sorbents had a similar Cu content (Table 3). The concentrations of Cu in the leachates were also similar and did not exceed limit values for waste being accepted at landfills (50 mg kg^−1^). The oxidisible Cu fraction was largest in the spent sorbents (Fig. 2), corresponding to approximately 70% of all the Cu.

Peat had a higher content of Zn compared with iron-peat (Table 3). A higher concentration of Zn was also present in the leachate from the iron-peat. It is possible that due to the Fe coating in the iron-peat, the organic groups involved in the adsorption of Zn were blocked (occupied) by Fe ions (Brown et al. 2000). Despite this, leaching of Zn from the iron-peat—as well as from the peat—was low and did not exceed limits for waste to be accepted at landfills for non-hazardous waste (50 mg kg^−1^). Similar to Cu, the oxidisible fraction of Zn was the largest, accounting for approximately 56% in both spent sorbents (Fig. 2).

After HTC treatment, approximately 15% of the Cu and 25% of the Zn were transferred to the process liquid from peat, and about 22% of Cu and 36% of Zn were transferred from the iron-peat. The sequential extraction revealed that the oxidisible fraction of Cu significantly decreased in the hydrochars (Fig. 2). Oxidisible fraction of Zn almost disappeared. The exchangeable fractions of Cu and Zn increased substantially. As a result, the concentrations of Cu in the hydrochar leachates were up to four times higher than in the spent sorbents leachates, and for Zn, this difference was up to 32 times. Divalent metals, such as Cu and Zn, usually form outer sphere complexes by reacting with hydroxylic and carboxylic groups of organic matter (Brown et al. 2000; Manceau and Matynia 2000). The FT-IR spectra (Fig. 1) revealed that these organic groups were primarily targeted during the hydrolysis of organic matter and were released into the process water. Due to the cleavage of easily oxygenated organic groups, it is likely that Cu and Zn were released into the process water either still bonded to low-molecular-weight compounds, such as monomeric alcohols and carboxylic acids, or as free ions that could later react with organic fragments and remain in solution (Berge et al. 2011).

### Treating spent sorbents

Landfilling waste with a high organic matter content can cause undesirable production of landfill gases that contribute to the greenhouse effect. Therefore, waste with more than 10% organic content is not accepted at European landfills. However, when waste does not fulfil the criteria outlined in the Council Decision 2003/33/EC (Annex II), and if there is no other viable utilisation option, landfilling can be allowed if a special permit is acquired. As well as with other wastes, leaching of metal(loid)s might become a limiting factor for waste acceptance. In the case of spent peat and iron-peat sorbents, neither could be accepted at landfills for hazardous waste. Leaching of As exceeded the limit values for peat, and leaching of Cr was above the limit in iron-peat. The spent sorbents would, therefore, require treatment prior to landfilling.

One of the reasons for applying HTC is the possibility of treating spent sorbents directly after contaminated water purification, while it is still wet. Additionally, carbonisation reactions and defragmentation of organic matter have been shown to improve the dewaterability of hydrochars (Libra et al. 2011). Although it was not evaluated in this study, such an improvement would be important on an industrial scale. More importantly, the potential to valorise hydrochar, as produced from spent sorbents (i.e. waste material that needs management), could be a notable improvement from the perspectives of socio-economics and circular economies.

However, the results obtained in this study show that HTC was ineffective at desorbing a sufficient proportion of metal(loid)s from spent sorbents to produce a clean hydrochar that could be used as a soil amendment without environmental risks. The treatment resulted in the process liquid and the hydrochar both having high loads of Cu, Zn, and, in particular, As. The leaching of As, Cu, and Zn from hydrochars was increased significantly compared with the spent sorbents, meaning that the hydrochars would not be accepted at landfills for hazardous waste without pre-treatment. Given the high caloric value of hydrochars, further management could be implemented via combustion. Although thermal treatment of As-rich waste is complicated because volatilisation of As can start at temperatures as low as 320 °C (Helsen et al. 2003), fabric filters and electrostatic precipitators (as are present in modern waste incineration plants) can remove more than 99% of particulate matter (Jones and Harrison 2016). Further management of the process liquid could also be implemented through various metal(loid) chemical precipitation methods (Fu and Wang 2011). Nevertheless, together these processes imply higher management costs for spent sorbents, meaning that direct combustion without HTC might be a better option. This would lead to a significantly reduced volume and mass of waste that needs to be managed.

In this study, the presence of As was a limiting factor for the reuse of the hydrochar. Arsenic does not belong to a group of essential microelements in soil, and applying As-containing materials back into the environment is not an option. Contrary to this, Cr, Cu, and Zn are essential micronutrients, and in the case of their depletion in soil, the development of plants might be disrupted. Therefore, the notion of producing hydrochar from specially designed spent sorbents should be further investigated. This would open up the possibility of improving soil fertility by simultaneously adding organic C and essential micronutrients.

## Conclusions

Peat-based spent sorbents underwent defragmentation of organic matter during HTC. Due to the cleavage of the easily oxygenated bonds, concentrations of low-molecular-weight fragments increased in the process liquid. Reducible fractions of As, Cu, and Zn decreased, while exchangeable fractions significantly increased in the obtained hydrochars. Consequently, large fractions of As, Cu, and Zn were transferred from the solids into the process water. However, the treatment was ineffective at desorbing a sufficient proportion of metal(loid)s (As in particular) from the spent sorbents to produce a clean hydrochar that could be used as a soil amendment without environmental risks. Due to the treatment, both process liquid and hydrochar became enriched with metal(loid)s meaning that further treatment of these two phases would be resource-demanding.

The leaching of As, Cu, and Zn from the hydrochars was significantly increased compared with the spent sorbents, which meant that the hydrochars would be unacceptable for disposal even in landfills designed for hazardous wastes.

Based on our results, the application of HTC to treat peat-based spent sorbents loaded with As, Cr, Cu, and Zn to produce a clean hydrochar for soil amelioration, or to ensure safe landfilling, does not appear to be feasible.
